# Thematic accuracy assessment of the NLCD 2019 land cover for the conterminous United States

**DOI:** 10.1080/15481603.2023.2181143

**Published:** 2023-03-01

**Authors:** James Wickham, Stephen V. Stehman, Daniel G. Sorenson, Leila Gass, Jon A. Dewitz

**Affiliations:** a Office of Research and Development, Environmental Protection Agency, Research Triangle Park, NC, USA; b College of Environmental Science and Forestry, State University of New York, Syracuse, NY, USA; c Western Geographic Science Center, U.S. Geological Survey, Seattle, WA, USA; d Western Geographic Science Center, U.S. Geological Survey, Tucson, AZ, USA; e Earth Resources and Observation Science (EROS) Center, U.S. Geological Survey, Sioux Falls, SD, USA

**Keywords:** Land cover change, MultiResolution Land Characteristics (MRLC) consortium, stratified random sampling, NLCD

## Abstract

The National Land Cover Database (NLCD), a product suite produced through the MultiResolution Land Characteristics (MRLC) consortium, is an operational land cover monitoring program. Starting from a base year of 2001, NLCD releases a land cover database every 2–3-years. The recent release of NLCD2019 extends the database to 18 years. We implemented a stratified random sample to collect land cover reference data for the 2016 and 2019 components of the NLCD2019 database at Level II and Level I of the classification hierarchy. For both dates, Level II land cover overall accuracies (OA) were 77.5% ± 1% (± value is the standard error) when agreement was defined as a match between the map label and primary reference label only, and increased to 87.1% ± 0.7% when agreement was defined as a match between the map label and either the primary or alternate reference label. At Level I of the classification hierarchy, land cover OA was 83.1% ± 0.9% for both 2016 and 2019 when agreement was defined as a match between the map label and primary reference label only, and increased to 90.3% ± 0.7% when agreement also included the alternate reference label. The Level II and Level I OA for the 2016 land cover in the NLCD2019 database were 5% higher compared to the 2016 land cover component of the NLCD2016 database when agreement was defined as a match between the map label and primary reference label only. No improvement was realized by the NLCD2019 database when agreement also included the alternate reference label. User’s accuracies (UA) for forest loss and grass gain were>70% when agreement included either the primary or alternate label, and UA was generally<50% for all other change themes. Producer’s accuracies (PA) were>70% for grass loss and gain and water gain and generally<50% for the other change themes. We conducted a post-analysis review for map-reference agreement to identify patterns of disagreement, and these findings are discussed in the context of potential adjustments to mapping and reference data collection procedures that may lead to improved map accuracy going forward.

## 1. Introduction

The United States National Land Cover Database (NLCD) (https://www.mrlc.gov) was initiated in the late 1990s (Loveland and Shaw 1996) to provide land cover for the nation from the Landsat satellites at their native spatial resolution of 30 × 30 m. The initiative has since grown into a land cover monitoring program that produces time-integrated (land cover at time *t* informs land cover at time *t-1*) land cover at 2- to 3-year intervals (Homer et al. 2020; Yang et al. 2018). The latest release, NLCD2019, includes land cover for 2001, 2004, 2006, 2008, 2011, 2013, 2016, and 2019. NLCD is recognized as a national geospatial data asset by the U.S. Federal Geographic Data Committee (FGDC 2021)

NLCD map production methodology is described thoroughly elsewhere (Homer et al. 2020; Jin et al. 2019; Yang et al. 2018). NLCD uses spectral (e.g. Landsat bands, indices), spatial (image objects), temporal (spectral succession and trajectory), and ancillary data as input into its land cover classification and post-classification processes. Random forest models are used for classification. Landsat 8 (launched 11 February 2013) images were used for the 2013, 2016, and 2019 dates of land cover in NLCD2019 and Landsat 5 was used for all earlier dates. Two of the main enhancements adopted for NLCD2019 production that were not incorporated in previous releases of NLCD were use of surface rather than top-of-the-atmosphere Landsat reflectance data (USGS 2021; 2022) and image composites. Use of surface rather than top-of-the-atmosphere reflectance corrects for atmospheric effects (Wulder et al. 2019). Image compositing follows from the widely utilized maximum Normalized Difference Vegetation Index (NDVI) methodology (Roy et al. 2010 and citations therein) first reported by Holben (1986). Compositing compares a suite of images over a given time period (e.g. season) and selects the “best” pixel on a per-pixel basis (Jin et al. in press; Qui et al. 2023). The method is based on the logic that the highest value is least corrupted by atmospheric effects and other factors and is also an efficient data reduction strategy (Holben 1986; Roy et al. 2010). NLCD image compositing (Jin et al. in press) was based on a median value concept (Flood 2013). This method provided superior change detection results when compared to a suite of image compositing methods (Qui et al. 2023). Image compositing was used to create leaf-on (May 1 to September 30) and leaf-off (November 1 to March 1) images for each date of land cover (Jin et al. in press).

Formal, statistically rigorous accuracy assessment is a prescribed component of NLCD because of its overarching monitoring objective (Homer et al. 2020; Yang et al. 2018) and its status as an operational program (Hansen and Loveland 2012). Accuracy assessment has followed all previous releases of NLCD (Stehman et al. 2003; Wickham et al. 2021, 2017, 2013, 2010). NLCD land cover data are used widely and therefore accuracy assessment is needed to support informed use of the data (Stehman and Czaplewski 1998). For example, NLCD is used to derive annual U.S. greenhouse gas emission estimates reported to the United Nations Framework Convention on Climate Change (UNFCCC) (EPA 2022). Accuracy assessment results also are used to improve the classification methods used for subsequent releases of NLCD data (Homer et al. 2020).

To denote the different versions of NLCD (based on date of release) and the year of land cover mapped, we use “NLCDxxxx” “yyyy” as terminology to describe the NLCD release date (“xxxx”) and the nominal date of land cover (“yyyy”). For example, NLCD2019 2013 would refer to the 2013 land cover in the NLCD2019 database. This terminology is identical to that used to report accuracies for NLCD2016 (Wickham et al. 2021). The release date (e.g. NLCD2019) refers to the nominal date of image acquisition, not the year the data were posted to the NLCD website (https://www.mrlc.gov). Each release of NLCD data includes updated versions of previous releases. NLCD2019 includes updated versions of each land cover date (2001, 2004, 2006, 2008, 2011, 2013, and 2016) released as part of NLCD2016. Prior accuracy assessments have focused on land cover released at a 5-year cycle; accuracy assessment of NLCD2016 focused on the nominal dates of 2001, 2006, 2011, and 2016. Here, we focus on the accuracy of the nominal dates of 2016 and 2019, reporting single-date and 2016–2019 change results for the NLCD2019 of the conterminous United States (i.e. United States, excluding Alaska and Hawaii). This is the first formal assessment of an NLCD interstitial date product and consequently provides an evaluation of accuracy of a 3-year change period versus the 5-year change periods of previous assessments.

## 2. Methods

The accuracy assessment methodology for NLCD2019 was nearly identical to that used for NLCD2016 (Wickham et al. 2021). The main differences were: 1) a reduction in the sample size to 3245 from 4629 used for NLCD2016, 2) inclusion of an additional stratum, and 3) a post-analysis review (Section 2.3) of factors that may have contributed to disagreement between map and reference labels. The reduction of the sample size, necessitated by programmatic changes, will reduce the precision of the accuracy estimators relative to the NLCD2016 assessment but the accuracy estimators will remain unbiased. However, because the standard errors of the accuracy estimates are directly related to the sample size and not the percent of the population sampled (Stehman 2001, 730), the sample size of 3245 is still large enough to yield acceptably small standard errors. The additional stratum was included to increase the sample size for changes from shrubland to grassland and vice versa. Grassland and shrubland comprise about 25% of the conterminous United States and change among these classes arises from ecological disturbances (e.g. fire), timber harvest and regeneration activities, and other agents of change. The post-analysis review was a systematic assessment of common error patterns which had not been undertaken in previous NLCD accuracy assessments. The main objectives of the post-analysis review were to inform future map production and future reference data collection.

### 2.1. Sampling design

Sample selection was based on 22 strata, defined by the NLCD2019 product, that included 15 no-change strata and 7 change strata. The 15 no-change strata were all NLCD Level II land cover classes (Table 1) except perennial ice and snow (class = 12), which was not included because it comprised ≪ 1% of the map (i.e. population). The stratification and sample size allocation were implemented primarily for the objective of estimating user’s accuracy of the rare land cover and change types. That is, the strata allowed us to increase the sample size for these rare classes to reduce the standard errors of the user’s accuracy estimates. The 7 change strata were:

1. Urban gain: not urban in 2016 and urban in 2019;
2. Forest loss: forest in 2016 and not forest in 2019, except for forest to urban (in urban gain stratum);
3. Forest gain: not forest in 2016 and forest in 2019;
4. Agriculture loss: agriculture in 2016 and not agriculture in 2019, except for agriculture to urban (in urban gain stratum) and agriculture to forest (in forest gain stratum);
5. Agriculture gain: not agriculture in 2016 and agriculture in 2019 except for forest to agriculture (in forest loss stratum);
6. Shrubland – grassland flux: shrubland in 2016 and grassland in 2019 or vice versa;
7. Catch-all – all 2016 to 2019 land cover changes not included in the other 6 strata. This stratum includes land cover changes at Level II that are not changes at Level I (e.g. pasture to cropland).

This sequence of steps for assigning each pixel to a stratum was necessary to guarantee that a pixel was assigned to one and only one stratum, as required of stratified sampling.

The 22 strata were created by combining the NLCD2019 2016 and 2019 land cover in a Geographic Information System (GIS) and assigning each pixel to the stratum to which it belonged (Table 2). The strata map was then disaggregated into individual (i.e. binary) maps which were used as a mask to generate a floating-point (double precision) random number map specific to each stratum. Pixels with random number values less than or equal to the ratio n/N, where n was the target sample size and N was the number of pixels in the stratum were selected for reference data collection. Like previous NLCD accuracy assessments, the strata map excluded coastal waters and the Great Lakes to avoid the likely reality that nearly all sample pixels in the water stratum would coincide with these water features. Nearly 4% of the Land Change Monitoring, Assessment, and Projection (LCMAP) program’s (Brown et al. 2020) initial sample locations (Pengra et al. 2020), for example, occurred in coastal waters or the Great Lakes because these features were not masked prior to sample selection.

### 2.2. Response design

Reference data collection followed the protocols established for previous NLCD accuracy assessments (Wickham et al. 2021). The main protocols were: 1) reference data collection by experienced interpreters (Bianchetti and MacEachren 2015); 2) blind interpretation (i.e. assignment of reference land cover labels without knowledge of the map label); 3) reliance on Google Earth^™^ imagery to address the accuracy assessment principle that the reference medium should be of higher quality than the mapping medium (Olofsson et al. 2014; Stehman and Foody 2019); 4) defining the pixel as the spatial support unit (Stehman and Wickham 2011); 5) reliance on image context (i.e. the neighborhood around the pixel) as a good practice to ensure correct reference label assignment (Stehman and Czaplewski 1998), and 6) use of primary and alternate reference labels to account for “fuzziness” of reference class determinations. Reference data labels for all 3,245 sample pixels were assigned by NLCD’s most experienced interpreter (protocol 1) using a blind format (protocol 2). The interpreter had participated in all NLCD accuracy assessments going back to NLCD2001 (Wickham et al. 2010). Reference label assignments were further supported by weekly web-enabled conference calls to discuss the most appropriate labels for a subset of the sample pixels. These pixels were selected because they represented difficult cases to classify. Nearly all coauthors participated in the weekly calls. Additional information on response design protocols is documented in the supplemental section of NLCD2016 accuracy assessment (Wickham et al. 2021)

Reference label assignment was based on interpretation of Google Earth^™^ imagery (protocol 3) for each selected sample pixel (protocol 4) by overlaying Keyhole Markup language Zipped (KMZ) files of points representing the center of the sample pixel, the polygon boundary of the 30 × 30 m sample pixel, and a 3-x-3 lattice of cells surrounding (and including) the sample pixel (protocol 5). The Google Earth^™^ platform includes an extensive collection of temporal, high-resolution imagery through agreements with several vendors. The image source and acquisition date are displayed in compliance with Google Earth^™^ protocols (Google Earth^™^ n.d.). Accompanying KMZ files have been made available (see Data Availability Statement) for those interested in viewing the reference data collected for this assessment.

Primary and alternate reference labels (protocol 6) have been used in all previous NLCD accuracy assessments (Stehman et al. 2003; Wickham et al. 2010, 2013, 2017, 2021) to address inherent class ambiguity (e.g. pasture versus grassland), geolocation errors , and mixed pixels. Use of two (primary and alternate) reference labels can be considered a special case of linguistic scale, fuzzy membership analysis (Stehman et al. 2003, 513) promoted by Gopal and Woodcock (1994). Consistent with accuracy assessment reporting for NLCD2011 (Wickham et al. 2017) and NLCD2016 (Wickham et al. 2021) error matrices are based on agreement between the map label and primary reference label only. User’s accuracy (UA), producer’s accuracy (PA), and overall accuracy (OA) are estimated from these error matrices. We also provide these accuracy estimates for the definition of agreement that allows a match with either the primary or alternate reference label. Each error matrix therefore includes two expressions of UA, PA, and OA (one for each definition of agreement). Alternate reference labels were used for 43% of the sample pixels at Level II and 28% of the sample pixels at Level I. For comparison, in the NLCD2016 accuracy assessment (Wickham et al. 2021) alternate reference labels were used for 63% of the sample pixels at Level II and 39% of the pixels at Level I.

For change accuracy, the definition of agreement when using the alternate reference labels did not include mixing of primary and alternate reference labels across dates. That is, agreement could occur if the primary reference labels for both 2016 and 2019 matched the map labels in 2016 and 2019, or if the alternate reference labels for both 2016 and 2019 matched map labels in 2016 and 2019. For example, suppose the NLCD2019 map labels were 50 (shrubland) for 2016 and 70 (grassland) for 2019, the primary reference labels were 50 for both 2016 and 2019, and the alternate reference labels were 50 for 2016 and 70 for 2019. For this example, there would be no agreement between map and reference labels when agreement was based on the primary reference label only, but there would be agreement when it was defined as a match between the map and either the primary or alternate reference label. Changing the example slightly, now suppose the NLCD2019 map labels were the same as defined previously (class 50 for 2016 and class 70 for 2019), but the primary reference labels were now 50 for both 2016 and 2019, and the alternate reference labels were 70 for both 2016 and 2019. For this example, there would not be agreement between the map and reference labels because neither the 2016–2019 set of primary labels (class 50 in 2016 and class 50 in 2019) nor the 2016–2019 set of alternate reference labels (70 and 70) matched the 2016–2019 set of map labels (50 and 70, respectively). For the assessment of binary change/no change accuracy, the first example would be considered a match only when agreement included that alternate reference label and the latter example would be considered disagreement regardless of agreement definition.

There was no available ca. 2016 Google Earth^™^ imagery for 164 sample pixels and nearly 43% (1385) of the sample pixels had no ca. 2019 Google Earth^™^ imagery. Only 56% (1814) of the sample pixels had Google Earth^™^ imagery for both 2016 and 2019; 118 sample pixels had no Google Earth^™^ imagery for either date. Landsat color composites were used as the reference medium when Google Earth^™^ imagery was unavailable. We evaluated the impact of missing Google Earth^™^ imagery on estimated accuracy for the single-date NLCD2019 2019 land cover and 2016–2019 binary change by comparing estimated accuracy for the two non-overlapping subsets of the sample, one with the Google Earth^™^ reference imagery and one without the requisite Google Earth^™^ reference imagery. We treated the two sample subsets as domains (SAS 2017, 9175) in the statistical assessment. For the single-date, NLCD2019 2019 comparison, there were 1860 sample pixels in the Google Earth^™^ imagery available domain and 1385 sample pixels in the Google Earth^™^ imagery missing domain. For the 2016–2019 comparison, there were 1822 sample pixels in the Google Earth^™^ imagery available domain and 1423 sample pixels in the Google Earth^™^ imagery missing domain. We did not assess the effect of missing Google Earth^™^ imagery on the single-date NLCD2019 2016 land cover because the relatively small percentage (5%) of missing ca. 2016 Google Earth^™^ imagery resulted in too few sample pixels to make the comparison meaningful.

### 2.3. Analysis

Analysis followed general estimation theory of probability sampling (cf. Särndal, Swensson, and Wretman 1992) that requires the inclusion probabilities derived from the stratified map (Section 2.2) (Stehman 2001; Stehman and Czaplewski 1998) and an indicator variable formulation of the estimators (Stehman 2014). Overall accuracy (OA) was estimated as:

(1) $$\hat{OA}=\frac{1}{N}\sum_{h=1}^{H}N_{h}{\hat{p}}_{h}$$

where ${\hat{p}}_{h}$ is the sample proportion of correctly classified pixels in stratum h, N is the total number of sample pixels, ${\text{N}}_{\text{h}}$ is the population size of stratum h, and the summation is over all H strata (H = 22). User’s accuracy (UA) and producer’s accuracy (PA) were expressed as a ratio, R=Y/X, where Y is the population total of ${\text{y}}_{\text{u}}$ and X is the population total of ${\text{x}}_{\text{u}}$, and ${\text{y}}_{\text{u}}$ and ${\text{x}}_{\text{u}}$ are indicator variables. For both user’s and producer’s accuracy of class K, ${\text{y}}_{\text{u}}=1$ when the map and reference labels are both class K, and ${\text{y}}_{\text{u}}=0$ otherwise. For user’s accuracy, ${\text{x}}_{\text{u}}=1$ if sample pixel u is map class K, and ${\text{x}}_{\text{u}}=0$ otherwise. For producer’s accuracy, ${\text{x}}_{\text{u}}=1$ if sample pixel u is reference class K, and ${\text{x}}_{\text{u}}=0$ otherwise. The combined ratio estimator (Cochran 1977, Section 6.11) for UA and PA is then:

(2) $$\hat{R}=\frac{\hat{Y}}{\hat{X}}=\frac{\sum_{h=1}^{H}N_{h}{\bar{y}}_{h}}{\sum_{h=1}^{H}N_{h}{\bar{x}}_{h}}$$

where ${\bar{x}}_{h}$ is the sample mean (proportion) of ${\text{x}}_{\text{u}}$ in stratum h and ${\bar{y}}_{h}$ is the sample mean of ${\text{y}}_{\text{u}}$ in stratum h. The estimated standard error of the combined ratio estimator is:

(3) $$SE\left(\hat{R}\right)=\sqrt{\frac{1}{{\hat{X}}^{2}}\sum_{h=1}^{H}N_{h}^{2}\left(1-\frac{n_{h}}{N_{h}}\right)\left(s_{yh}^{2}+{\hat{R}}^{2}s_{xh}^{2}-2\hat{R}s_{xyh}\right)/n_{h}}$$

where ${\text{n}}_{\text{h}}$ is the sample size in stratum h, $s_{yh}^{2}$ and $s_{xh}^{2}$ are The review was a systematic examination of selected the sample variances for ${\text{y}}_{\text{u}}$ and ${\text{x}}_{\text{u}}$ in stratum h, and $s_{xyh}$ is the sample covariance for ${\text{y}}_{\text{u}}$ and ${\text{x}}_{\text{u}}$ in stratum h, and

(4) $$\hat{X}=\sum_{h=1}^{H}N_{h}{\bar{x}}_{h}$$

is the estimated population total of ${\text{x}}_{\text{u}}$. Estimates were computed using SAS (version 9.4) (SAS Institute Inc 2017).

Error matrices, with the reference labels forming the columns and the map labels forming the rows, were used to report results. Cell entries are the estimated percent of area for the conterminous United States (i.e. map area) for each realized combination of map and reference label. Multiplying the area area of the conterminous US (7,789,691 km^2^ from Table 2), by a cell proportion estimates the area labeled as map class = A and reference class = B. UA and PA for both definitions of agreement – map label matches primary reference label only and map label matches either primary or alternate reference label – are reported with the error matrices. Estimates of map bias, calculated as map class area proportion minus reference class area proportion (Olofsson et al. 2014) are also reported for each error matrix.

### 2.4. Post-analysis review

The post-analysis review was a reexamination of reference and map labels following completion of accuracy estimation. The purpose of the review was to uncover error patterns that could be used to inform future NLCD land cover products and accuracy assessments. The results from the post-analysis review were not used in any of the accuracy results reported herein. The review, conducted by three of the coauthors, focused on error patterns in most of the change strata and the open urban class (Level II code = 21). sample pixels that had mismatched map and reference labels, supported by Google Earth^™^ imagery, Landsat color composites and the land cover map itself (Table 3). The post-analysis review focused primarily on the land cover change strata to further support the land cover change monitoring objective of NLCD (Homer et al. 2020; Yang et al. 2018). The Level II open urban class was included because of its consistently high commission error reported in previous NLCD accuracy assessments (e.g. Wickham et al. 2021)

## 3. Results

### 3.1. Single-date accuracy

At Level II of the classification hierarchy, OA for both NLCD2019 2016 and NLCD2019 2019 were 77.5% ± 1.0% (± value is the standard error) when agreement was based on a match between the map label and primary reference label only and improved to 87.1% ± 0.7% when agreement also included the alternate reference label (Tables 4 and 5). Based on the primary only agreement definition, the OA for NLCD2019 2016 represents a substantial improvement of 5% over that reported for NLCD2016 2016 (Wickham et al. 2021). The 5% improvement in OA represents an increase of about 390,000 km^2^ in area that is accurately mapped in NLCD2019 2016 compared to NLCD2016 2016. The substantial increase in NLCD2019 2016 OA relative to NLCD2016 2016 OA did not hold when agreement also included the alternate reference label as the OA for NLCD2016 2016 was 86.9% (Wickham et al. 2021) compared to 87.1% for NLCD2019 2016. At Level I of the classification hierarchy, OA was 83.1% ± 0.9% when a match included only the primary reference label for the 2016 and 2019 land cover dates in NLCD2019 and improved to 90.3% ± 0.7% when agreement also included the alternate reference label (Tables 6 and 7). Similar to Level II, the Level I OA of 83.1% for NLCD2019 2016 for the primary only definition of agreement was a 4% improvement in Level I OA when compared to NLCD2016 2016 for the same agreement definition.

Missing Google Earth^™^ reference imagery appeared to have a counterintuitive effect on NLCD2019 2019 accuracy (Table 8). That is, human interpretation of Landsat (reference classification) tended to have higher agreement with the NLCD (machine) classification of Landsat relative to human interpretation of Google Earth^™^ compared to the NLCD classification of Landsat (i.e. the with Google Earth^™^ subset). OA was about 6% higher for the subset missing Google Earth^™^ reference imagery than the subset with Google Earth^™^ reference imagery regardless of agreement definition. The differences in OA between the subsets were also evident in the UA and PA values. Overall, UA and PA tended to be higher for the subset of sample pixels that did not have 2019 Google Earth^™^ reference imagery compared to the subset that had 2019 Google Earth^™^ reference imagery. UA for the medium- and high-density urban classes (Level II = 23 and 24) were exceptions to this tendency. UA for the with Google Earth^™^ subset had much higher UA values for these two classes than the without Google Earth^™^ subset. The high PA values for classes 23 and 24 in the without Google Earth^™^ subset were likely attributable to very low sample counts, which were<10 for both classes.

### 3.2. Binary and theme-specific change accuracy

Accuracy of binary change versus no change was consistent with the previous NLCD2016 accuracy assessment (e.g. Wickham et al. 2021). OA was 83% when agreement was defined as a match between the map label and only the primary reference label and improved to 90% when agreement also included the alternate reference label (Table 9). OA reflects the dominance and high UA and PA for the no change class, as UA and PA of no change were>95% regardless of agreement definition. UA and PA for the change class were 41% and 42%, respectively, when agreement included only the primary reference labels and improved to 48% and 63%, respectively, when the alternate reference label was also included in the agreement definition.

The effect of missing Google Earth^™^ reference imagery on binary change was similar to the result for single-date NLCD2019 2019 land cover. Regardless of agreement definition, change UA was nearly identical across the with and without Google Earth^™^ domains, and hence nearly equivalent the UA values reported in Table 9 for the two agreement definitions. In contrast, binary change PA was higher for the without Google Earth^™^ domain than the with Google Earth^™^ domain. However, SE increased substantially for the without Google Earth^™^ domain indicating that the differences may be within the range of sampling variability of the PA estimates of the two domains.

Previous NLCD accuracy assessments have reported UA and PA for loss and gain themes for water, urban (gain only), forest, shrub, grass, and agriculture based on agreement between the map label and either the primary or alternate reference labels (Wickham et al. 2021, Table 10). Using the same agreement definition, loss and gain UA and PA for each of these themes was about the same for NLCD2019 2016–2019 change compared to NLCD2016 2011–2016 change reported in Wickham et al. (2021), although there were slightly more substantial declines than there were increases in UA and PA. UA and PA for the NLCD2019 2016–2019 no change classes improved slightly compared to their NLCD2016 2011–2016 counterparts. Urban gain UA and PA for NLCD2019 were very low and much less than previous NLCD accuracy assessments. Forest loss UA was consistent with that reported for 2011–2016 but forest loss PA was lower.

Not surprisingly, the low UA and PA across loss and gain themes were dominated by confusion with the theme-specific no change and the theme-specific never (e.g. never urban) categories of the 4-x-4 change error matrices. For example, the dominant source of urban gain omission error was area mapped as never urban (Table 11), and similarly the largest source of forest gain omission error was area mapped as never forest (Table 12). The primary source of forest gain commission error was area of stable forest that was incorrectly mapped as forest gain (Table 12). The never category was almost exclusively the highest off-diagonal cell entry for both commission and omission error for the other change themes (results not shown).

### 3.3. Post-analysis review

Several error patterns were uncovered as a result of conducting the post-analysis review.

- Urban gain accuracy was impacted by road data error. Disagreement for 13 of the 35 sample pixels examined appeared to be attributable to road data error. Roads that were not constructed between ca. 2016 and ca. 2019 based on examination of temporal Google Earth^™^ imagery were identified as new road construction between ca. 2016 and ca. 2019 by NLCD. These sample pixels tended to occur in remote areas that had small, often unpaved roads, or no roads (i.e. not visible on Google Earth^™^ imagery).
- Forest loss accuracy did not show any strongly discernable patterns in the post-analysis review. In most cases a loss in vegetation biomass was not apparent in the Google Earth^™^ imagery. At a continental scale, there was a much higher rate of forest loss map-reference agreement in the eastern U.S. than the western U.S.
- Forest gain accuracy did not show any strongly discernable patterns in the post-analysis review. In most cases, an increase in vegetation biomass was not apparent in the Google Earth^™^ imagery. Consequently, reference labels for sample pixels mapped as forest gain most often indicated that change did not occur. The geographic pattern of the sample pixels mapped as forest gain, being concentrated in the Pacific Northwest and southeast, appeared to be strongly associated with timber harvest and regeneration activities. There was a much higher likelihood of forest gain map-reference agreement in the southeast than elsewhere.
- Agriculture loss error revealed no discernable patterns in the post-analysis review. More broadly, reference labels for sample pixels mapped as agriculture loss stratum almost always indicated no change. Reference labels in this stratum that indicated change comprised only 20% of the sample pixels mapped as agriculture loss.
- Agriculture gain error was similar to agriculture loss. Most sample pixels mapped as agriculture gain had reference labels indicating no change. The main difference in the error patterns between agriculture loss and agriculture gain was the spread across the 8 no-change, Level I classes. For agriculture gain, almost all reference labels indicated static agriculture, whereas the reference labels for agriculture loss indicated no change spread more evenly across urban, barren, agriculture, and wetland.
- Shrubland-grassland flux error appeared to be associated with aridity. Sample pixels that coincided with low, sparse shrub cover, as depicted in the Google Earth^™^ imagery, tended to have mismatched map and reference labels. For example, about dozen sample pixels mapped as shrubland-grassland flux were in west Texas and New Mexico. Nearly all had mismatched map and reference labels and the vegetation cover apparent in the Google Earth^™^ imagery tended to be a sparse coverage of small shrubs punctuating a more widespread coverage of grass. Agreement was much higher in the eastern U. S., especially the southeast, and associated with apparent tree harvesting and regeneration activities. Nearly 70% of the sample pixels of this type of mapped change located east of the Mississippi River had matching map and reference labels.
- Open urban was confounded by class ambiguity and the presence of remote roads. Upon reexamination of the map and reference labels, it was found that a case could be made for assigning map-matching reference labels (primary or alternate) for 20 of the 50 sample pixels examined. For the sample pixels that had 81 (pasture) or 71 (grassland) as the map label and open urban as the primary reference label, disagreement appeared to be a matter of context. These sample pixels tended to occur in rural areas of the Midwest and Great Plains coincident with agricultural buildings. Ancillary data used to model impervious area include these isolated structures but not the yards and managed vegetation around them. For the sample pixels that had forest (41, 42, or 43) as the map label and open urban as the primary reference label, disagreement appeared to be a lack of clarity on the class of road included in the models used to produce NLCD land cover. Many of these sample pixels were in remote areas, often associated with apparent tree harvesting and regeneration activities that had unpaved roads within or adjacent to the sample pixel.

## 4. Discussion

Accuracy of NLCD2019 was consistent with previous releases of NLCD (Wickham et al. 2021). Level II and Level I single-date accuracies were 87% and 90%, respectively, when the alternate reference label was included in the definition of agreement. Change accuracies were also consistent with previous assessments of NLCD land cover change. Improvements in the precision of change PA estimates may have been realized if the sample size allocation to strata were tailored to mitigate the effect of the area dominance of the no change class (Olofsson et al. 2020; Stehman and Foody 2019). That is, the standard error of the PA estimate is reduced when larger sample sizes are allocated to the common (larger) strata. However, a primary motivation of the stratified design was to achieve adequately small standard errors for UA of rare classes, which requires increasing the sample size for rare classes. Therefore, precise estimation of PA and UA are competing objectives in terms of how to allocate the sample to strata. The only way to resolve the conflict would have been to increase the total sample size and allocate these additional sample pixels to the common strata. Unfortunately, programmatic constraints could not accommodate adding additional accuracy assessment objectives beyond optimizing for small UA standard errors for rare classes.

The post-analysis review, adopted as an additional accuracy assessment protocol new to NLCD, revealed error patterns that may lead to improvements in land cover production and reference data collection. Identification of road data error in urban gain sample pixels provides information that may be useful to NLCD map producers. Editing of road data is a component of NLCD production (Homer et al. 2020). Because of program changes, NLCD lost access to the GIS road dataset used to produce NLCD2011 and NLCD2016. Changes in GIS road data sources may have contributed to the attribution of roads as newly constructed between ca. 2016 and ca. 2019, which likely reduced urban gain accuracy. The error pattern in the shrubland – grassland flux stratum suggests that methods for classifying change between shrubland and grassland could be refined. Temporal NDVI patterns or products from other MRLC members (Wickham et al. 2014) could be incorporated into the modeling process, which might lead to improved accuracy of mapped changes from shrubland to grassland and vice versa. Likewise, the error patterns in the agriculture gain and loss change themes also suggest additional post processing could improve the accuracy of these strata. Post processing could include a direct comparison with map labels from the annually produced Cropland Data Layer (CDL) (Boryan et al. 2011). CDL reports high thematic accuracy for major crop types such as corn, soybeans, and cotton that comprise the majority of area devoted to crops (Boryan et al. 2011; Lark, Schelly, and Gibbs 2021).

The post-analysis review also highlighted areas where NLCD reference label protocols could be improved. Particularly, uncertainty regarding whether the presence of a road should factor into the reference classification (e.g. Level II class = 21) appears to have contributed to mismatched map and reference labels. Unpaved roads are also a frequent source of uncertainty. The response design protocols (Stehman and Czaplewski 1998) developed and used by NLCD (Wickham et al. 2021, Supplemental Information) did not include guidelines for when and how roads should factor into the reference label assignment. These guidelines could be updated to include more specific definitions for inclusion of roads in the developed (urban) classes.

We were surprised by the counterintuitive impact of reference imagery availability on accuracy results. We would have expected lack of Google Earth^™^ imagery to have a dampening effect on accuracy. Reliance on reference data that are of higher quality than the data used to derive the map classification is a fundamental principle of accuracy assessment (Olofsson et al. 2014; Stehman and Foody 2019). Higher quality is most commonly interpreted as higher spatial resolution but guidance for using Landsat images as reference data has also been articulated (Olofsson et al. 2014; Stehman and Foody 2019). We speculate that experience may have been an influential factor contributing to the counterintuitive results. The reference classification was done by the NLCD project’s most experienced interpreter, who has contributed to or led reference data collection for all previous NLCD accuracy assessments. Experience is recognized as an important factor in the collection of accurate reference classification data (Bianchetti and MacEachren 2015; Van Coillie et al. 2014). However, we are not aware of any studies examining interpreter experience as a factor mitigating the potential negative influence of poor quality or missing reference imagery. NLCD reference data has, as a standard of practice, included a “notes” field, which is used to record the context of sample pixels and any interpretation challenges (e.g., uncertainty in reference label assignment). We searched the “notes” field of the 1431 sample pixels that had missing 2016 or 2019 Google Earth^™^ imagery for phrases indicating missing Google Earth^™^ imagery complicated reference label assignment (e.g. “hard to tell without 2019 Google Earth^™^ imagery”). Only 41 of the 1431 (3%) sample pixels with missing 2016 or 2019 Google Earth^™^ imagery included such phrases, suggesting the missing Google Earth^™^ imagery made reference label assignment a challenge only rarely. More precisely, our speculation is that with sufficient experience in the tandem comparison of high-resolution and Landsat imagery to collect reference classification data, the hinderance imposed by the absence of the high-resolution component of the tandem becomes less challenging because of the wealth of information in the mental database accrued through all previous efforts.

## 5. Summary and conclusion

Overall, NLCD2019 land cover thematic accuracy results are consistent with assessment of previous NLCD land cover accuracy assessments. Single-date level II and Level I overall accuracies for NLCD2019 2016 improved measurably compared to their counterparts reported for NLCD2016 2016 (Wickham et al. 2021). Differences in UA and PA for loss and gain change themes between NLCD2019 2016–2019 and NLCD2016 2011–2016 were equivocal. Accuracy of NLCD2019 binary change for 2016–2019 was nearly identical to NLCD2016 binary change accuracy for 2011–2016 (Wickham et al. 2021; Table 10), and there was a mix of substantial UA and PA increases and decreases across the loss and gain change themes when compared to the previous NLCD land cover accuracy assessment (Table 10). The history of NLCD accuracy assessment, to which this article contributes, indicates that NLCD produces highly accurate single-date land cover maps at Level II and Level I, but similarly accurate mapping of land cover change continues to be elusive. Only forest loss user’s accuracy has been consistently≥70% across all NLCD land cover change accuracy assessments (Wickham et al. 2013, 2017, 2021). Protocols such as the post-analysis review of map errors may contribute to the ongoing effort to improve NLCD change accuracy.

## Figures and Tables

**Table 1.** NLCD 2019 land cover legend (see, USGS n.d., in citations for a more detailed description).

Land Cover Class	Level II code	Level I code	Description
Open Water	11	10	Open water, < 25% vegetation or bare soil
Perennial Ice/Snow	12	10	At least 25% year-round ice or snow
Developed, open space	21	20	impervious cover (IC) < 20%
Developed, low intensity	22	20	20% ≤ IC < 50%
Developed, medium intensity	23	20	50% ≤ IC < 80%
Developed, high intensity	24	20	IC ≥ 80%
Barren land	31	30	Areas of bedrock, vegetation<15%
Deciduous forest	41	40	≥20% WV ≥5 m & ≥ 75% sheds foliage seasonally
Evergreen forest	42	40	≥20% WV ≥5 m & ≥ 75% with persistent foliage
Mixed forest	43	40	≥20% WV ≥5 m & neither D nor E ≥ 75%
Shrubland	52	50	≥20% WV <5 m
Grassland	71	70	≥80% herbaceous, management not apparent
Pasture/hay	81	80	≥20% herbaceous, managed for livestock, hay, other
Cropland	82	80	≥20% herbaceous, managed for annual crops
Woody wetland	90	90	≥20% WV on periodically saturated soils
Herbaceous wetland	95	90	≥80% perennial herbaceous on periodically saturated soils

Abbreviations: 5 m = 5 meters in height; D = deciduous; E = Evergreen; IC = impervious cover; WV = woody vegetation.

**Table 2.** Strata for the sampling design of the NLCD2019 accuracy assessment. Except for stratum 16, the change strata are resolved at Level I of the classification hierarchy (see Table 1). The Level II perennial ice and snow land cover class and land cover changes at Level II of the classification hierarchy but not at Level I were included in stratum 16. The area of the population (map) is 7,789,691 km^2^ (8,665,213,260 pixels).

Stratum	# pixels	%	Description
1	147,082,445	1.70	Water, no change (does not include perennial ice & snow)
2	238,486,918	2.76	Open space developed, no change
3	151,239,589	1.75	Low-density developed, no change
4	88,083,025	1.02	Medium-density developed, no change
5	31,595,484	0.37	High-density developed, no change
6	83,865,051	0.97	Barren, no change
7	821,080,287	9.49	Deciduous forest, no change
8	995,731,978	11.50	Evergreen forest, no change
9	298,680,746	3.45	Mixed forest, no change
10	1,868,977,201	21.59	Shrubland, no change
11	1,141,536,779	13.19	Grassland, no change
12	558,882,589	6.46	Hay/pasture, no change
13	1,458,350,116	16.85	Cropland, no change
14	399,595,939	4.62	Woody wetland, no change
15	127,911,404	1.48	Emergent wetland, no change
16	28,322,137	0.33	Catch all: all perennial ice & snow and 2016–2019 change pixels not in strata 17–22
17	4,377,737	0.05	Urban gain: not urban in 2016 and urban in 2019
18	41,314,470	0.48	Forest loss: forest in 2016 and not forest in 2019 except for forest to urban (stratum 17)
19	56,483,220	0.65	Forest gain: not forest in 2016 and forest in 2019
20	1,385,948	0.02	Agriculture (Ag) loss: Ag in 2016 and not Ag in 2019 except for Ag to forest (Stratum 19) and Ag to urban (stratum 17)
21	7,403,458	0.09	Ag gain: not Ag in 2016 and Ag in 2019 except for forest to Ag (stratum 18)
22	104,826,739	1.21	Shrub-grass flux: shrubland in 2016 and grassland in 2019 or vice versa
Total	8,655,213,260		

**Table 3.** Strata from which sample pixels were selected for post-analysis review.

Stratum	Description
Urban gain	Examination of 35 sample pixels that changed from non-urban to urban between 2016 and 2019
Forest loss	Examination of 70 sample pixels that changed from forest to non-forest between 2016 and 2019
Forest gain	Examination of 70 sample pixels that changed from non-forest to forest between 2016 and 2019
Agriculture loss	Examination of 20 sample pixels that changed from agriculture to non-agriculture between 2016 and 2019
Agriculture gain	Examination of 20 sample pixels that changed from non-agriculture to agriculture between 2016 and 2019
Shrub-grass flux	Examination of 50 sample pixels that changed from shrubland to grassland between 2016 and 2019 and vice versa
Open urban (Level II code = 21)	Examination of 50 sample pixels that had 21 as the map label and 81 (pasture), 71 (grassland), or forest (41, 42, or 43) as primary reference label

**Table 4.** Level II agreement between map and reference labels for NLCD2019 2019 for the conterminous United States. Class codes are in Table 1. Cell entries are percentage of map area (7,789,691 km^2^; see also Table 2 caption). OA, PA, and UA are overall, producer’s, user’s accuracies (and their standard errors) based on agreement defined as the map label matching the primary label only. OAa, PAa, and UAa, are the corresponding estimates for agreement defined as either the primary or alternate reference labels matching the map label. SE is the standard error of the class areas estimates based on the reference (column) label. Map bias (MB) is row (map) total minus reference (column) total. MB is the error attributable to estimating class area based on the map (i.e.; pixel counting), as discussed in Olofsson et al. (2014). Sum of MB ≠ 0 due to rounding error. Accuracy estimates (e.g. UA, UAa) and their (SE) are rounded to the nearest integer. OA = 77.5% ± 1.0%; OAa = 87.1% ± 0.7%.

	Reference																				
Map	11	12	21	22	23	24	31	41	42	43	52	71	81	82	90	95	Total	UA	UAa	MB	n
11	**1.6970**		0.0227				0.0028				0.0009	0.0009	0.0009	0.0022	0.0227	0.0062	1.7562	97 (2)	100 (0)	−0.0078	150
12		**0.0018**					0.0018										0.0035	50 (25)	75 (22)	0.0017	4
21			**1.9974**	0.3594	0.0555			0.0567	0.0563	0.0284	0.1122	0.0008	0.0843	0.0295			2.7805	72 (4)	88 (3)	−1.9365	163
22			0.7437	**0.6857**	0.2472	0.0183		0.0349			0.0175	0.0004	0.0004	0.0192			1.7673	39 (5)	69 (5)	0.4759	133
23			0.1800	0.2144	**0.5017**	0.1454	0.0009	0.0018			0.0102	0.0106		0.0127			1.0777	47 (5)	64 (5)	0.2011	186
24			0.0377	0.0310	0.0591	**0.2404**	0.0073	0.0004	0.0037							0.0037	0.3831	63 (5)	72 (4)	−0.0210	127
31	0.0267		0.0066	0.0009	0.0131		**0.6464**	0.0022	0.0150		0.2106	0.0732	0.0006	0.0015		0.0129	1.0098	64 (5)	75 (5)	0.2015	156
41			0.1265				0.0632	**7.4923**	0.0748	1.0867	0.3336	0.1294	0.1265		0.1265	0.0661	9.6258	78 (3)	86 (3)	−0.4904	198
42			0.2001				0.0029	0.0686	**10.6498**	0.2804	0.5066	0.2659					11.9743	89 (3)	94 (2)	−1.0402	337
43			0.1725					0.4486	0.3596	**2.2002**	0.0374	0.1035	0.1380		0.0345		3.4944	63 (5)	74 (4)	−0.7371	115
52			0.2373				0.0812	0.2076	0.8075	0.0905	**18.1278**	2.6202	0.3345	0.1688			22.6753	80 (2)	92 (2)	1.3757	421
71			0.2138				0.0009	0.1483	0.3855	0.0680	1.6320	**9.9848**	0.7924	0.3336	0.0009	0.2655	13.8258	72 (3)	88 (2)	0.0266	454
81			0.5173					0.3889	0.0646		0.1299	0.3229	**4.5290**	0.4527		0.0646	6.4698	70 (5)	79 (4)	−0.2797	117
82			0.1700					0.3377	0.0842		0.0865	0.1759	0.5169	**15.3853**	0.0842	0.0850	16.9258	91 (2)	94 (2)	0.4747	302
90			0.0471					0.8810	0.5135	0.4617	0.0640	0.0479	0.0477	0.0002	**2.5105**	0.0965	4.6700	54 (5)	60 (5)	1.6303	175
95	0.0403		0.0443				0.0009	0.0470		0.0157	0.0304	0.0629	0.1784	0.0452	0.2604	**0.8352**	1.5608	54 (5)	63 (5)	0.1251	207
Total	1.7640	0.0018	4.7170	1.2914	0.8766	0.4041	0.8083	10.1162	13.0145	4.2315	21.2996	13.7992	6.7495	16.4511	3.0397	1.4357					
SE	0.0426	0.0012	0.3616	0.1334	0.0904	0.0434	0.1144	0.4805	0.4462	0.3699	0.6555	0.6409	0.4723	0.4240	0.2709	0.2057					
PA	96 (1)	100 (0)	42 (3)	53 (5)	57 (5)	59 (6)	80 (10)	74 (3)	82 (2)	52 (4)	85 (2)	72 (3)	67 (4)	94 (1)	83 (5)	58 (8)					
PAa	98 (1)	100 (0)	59 (4)	88 (2)	81 (5)	82 (7)	83 (9)	84 (2)	87 (2)	67 (5)	95 (1)	86 (2)	79 ((3)	95 (1)	90 (3)	68 (8)					
n	147	2	292	113	113	98	90	240	395	124	438	472	169	309	112	131					3245

**Table 5.** Level II agreement between map and reference labels for NLCD2019 2016 for the conterminous United States. See Table 4 for an explanation of contents. Sum of MB ≠ 0 due to rounding error. OA = 77.6% ± 1.0%; OAa = 87.2% ± 0.7%.

	Reference																				
Map	11	12	21	22	23	24	31	41	42	43	52	71	81	82	90	95	Total	UA	UAa	MB	n
11	**1.6491**		0.0227				0.0018	0.0009				0.0044		0.0009	0.0280	0.0457	1.7533	94 (2)	99 (0)	0.0316	136
12		**0.0018**					0.0018										0.0035	50 (25)	75 (22)	0.0017	4
21			**2.0175**	0.3911	0.0320			0.0560	0.2690	0.2760	0.1102		0.0835	0.0320			2.8067	72 (4)	89 (3)	−1.8947	158
22			0.7545	**0.6859**	0.2473	0.0175		0.0367			0.0175			0.0175			1.7677	39 (5)	69 (5)	0.4487	123
23			0.1527	0.2137	**0.4783**	0.1332					0.0204	0.0102		0.0102			1.0186	47 (5)	65 (5)	0.2826	101
24			0.0365	0.0256	0.0548	**0.2236**	0.0073		0.0037							0.0037	0.3650	64 (5)	73 (4)	−0.0197	100
31	0.0338		0.0022	0.0004	0.0129		**0.6492**	0.0018			0.2294	0.0787	0.0018	0.0035	0.0009	0.0165	1.0310	63 (5)	74 (5)	0.2168	144
41			0.1281			0.0004	0.0632	**7.4392**	0.0720	1.0797	0.3225	0.1328	0.1265		0.1265	0.0632	9.5541	78 (3)	87 (3)	−0.5107	192
42			0.1988				0.0042		**10.5513**	0.3375	0.5469	0.2182		0.0021			11.8588	89 (2)	95 (2)	−1.1165	354
43			0.1754					0.4561	0.3943	**2.2112**	0.0736	0.0366	0.1380		0.0345		3.5198	63 (5)	73 (3)	−1.2309	141
52	0.0027		0.2531				0.0812	0.2332	0.8498	0.1307	**17.9538**	2.4732	0.3165	0.1608		0.0018	22.4567	80 (2)	92 (1)	1.2748	569
71	0.0009		0.2040	0.0004	0.0004		0.0027	0.1217	0.4239	0.0785	1.7371	**10.3575**	0.8280	0.2996		0.2693	14.3240	72 (3)	88 (2)	0.3335	452
81	0.0005		0.4560	0.0016			0.0011	0.4526	0.0649	0.0002	0.0656	0.3891	**4.5921**	0.3899	0.0005	0.0658	6.4795	71 (5)	79 (4)	−0.2556	192
82			0.1711		0.0004		0.0009	0.3373	0.0842		0.0850	0.1688	0.4220	**15.4237**	0.0847	0.0844	16.8625	91 (1)	95 (2)	0.4745	257
90			0.0923	0.0004				0.8825	0.4709	0.5105		0.0471	0.0466	0.0011	**2.5055**	0.0932	4.6501	54 (5)	60 (5)	1.6069	140
95	0.0349		0.0456				0.0009	0.0470	0.0035	0.0148	0.0201	0.0740	0.1801	0.0467	0.2627	**0.8182**	1.5486	53 (5)	64 (5)	0.0868	182
Total	1.7217	0.0018	4.7014	1.3190	0.8260	0.3847	0.8142	10.0648	12.9753	4.3907	21.1819	13.9905	6.7351	16.3880	3.0432	1.4618					
SE	0.0478	0.0012	0.3595	0.1357	0.0862	0.0424	0.1145	0.4753	0.4320	0.3778	0.6465	0.6365	0.4633	0.4074	0.2709	0.2070					
PA	96 (2)	100 (0)	42 (3)	52 (5)	58 (6)	61 (6)	80 (10)	74 (3)	81 (2)	50 (4)	85 (2)	74 (3)	68 (4)	94 (1)	82 (4)	56 (8)					
PAa	98 (1)	100 (0)	60 (4)	87 (3)	83 (5)	82 (7)	83 (9)	83 (2)	87 (2)	65 (5)	95 (1)	88 (2)	80 (3)	95 (1)	91 (3)	67 (8)					
n	118	2	286	102	88	80	95	254	488	153	475	369	181	303	116	135					

**Table 6.** Level I agreement between map and reference labels for NLCD2019 2019. See Table 4 for an explanation of contents. Sum of MB ≠ 0 due to rounding error. OA = 83.1% ± 0.9%; OAa = 90.3% ± 0.7%.

	Reference													
Map	10	20	30	40	50	70	80	90	Total	UA	UAa	MB	n
10	**1.6996**	0.0227	0.0037		0.0009	0.0009	0.0031	0.0288	1.7597	97 (2)	100 (0)	−0.0070	154
20		**5.4994**	0.0082	0.1821	0.1399	0.0118	0.1462	0.0211	6.0086	92 (2)	94 (1)	−1.2630	609
30	0.0267	0.0205	**0.6464**	0.0173	0.2106	0.0732	0.0021	0.0129	1.0098	64 (5)	75 (5)	0.2024	156
40		0.4992	0.0661	**22.6611**	0.8776	0.4988	0.2645	0.2271	25.0944	90 (1)	94 (1)	−2.3337	650
50		0.2373	0.0812	1.1055	**18.1278**	2.6022	0.5033		22.6753	80 (2)	91 (2)	1.3757	421
70		0.2138	0.0009	0.6678	1.6320	**9.9189**	1.1259	0.2664	13.8258	72 (3)	88 (2)	0.0925	454
80		0.6873		0.8755	0.2164	0.4988	**20.8839**	0.2338	23.3956	89 (2)	92 (1)	0.1950	419
90	0.0403	0.0914	0.0009	1.9188	0.0945	0.1108	0.2714	**3.7026**	6.2308	59 (4)	65 (4)	1.7379	382
Total	1.7667	7.2716	0.8074	27.4281	21.2996	13.7333	23.2006	4.4929	100.0000				
SE	0.0426	0.3420	0.1144	0.5952	0.6555	0.6424	0.5926	0.3273					
PA	96 (2)	76 (3)	80 (10)	83 (1)	85 (2)	72 (3)	90 (1)	82 (4)					
PAa	98 (1)	83 (3)	83 (9)	88 (1)	95 (1)	87 (2)	93 (1)	90 (4)					
n	150	615	89	760	438	471	478	244					3245

**Table 7.** Level I agreement between map and reference labels for NLCD2019 2016. See Table 4 for an explanation of contents. Sum of MB ≠ 0 due to rounding error. OA = 83.1% ± 0.9%; OAa = 90.5% ± 0.7%.

	Reference													
Map	10	20	30	40	50	70	80	90	Total	UA	UAa	MB	n
10	**1.6508**	0.0227	0.0035	0.0009		0.0044	0.0009	0.0736	1.7568	94 (2)	99 (0)	0.0333	140
20		**5.4649**	0.0073	0.1808	0.1480	0.0102	0.1432	0.0037	5.9580	92 (2)	94 (1)	−1.2732	482
30	0.0338	0.0155	**0.6492**	0.0018	0.2294	0.0787	0.0053	0.0173	1.0310	63 (5)	74 (5)	0.2168	144
40		0.5027	0.0674	**22.5412**	0.9430	0.3875	0.2666	0.2242	24.9327	90 (1)	94 (1)	−2.4981	687
50	0.0027	0.2531	0.0812	1.2137	**17.9545**	2.4732	0.4765	0.0018	22.4567	80 (2)	92 (2)	1.2741	569
70	0.0009	0.2048	0.0027	0.6241	1.7371	**10.3575**	1.1276	0.2693	14.3240	72 (3)	88 (2)	0.3335	452
80	0.0005	0.6291	0.0020	0.9391	0.1505	0.5579	**20.8277**	0.2354	23.3420	89 (2)	93 (1)	0.2197	449
90	0.0349	0.1384	0.0009	1.9292	0.0201	0.1211	0.2745	**3.6797**	6.1987	59 (4)	65 (4)	1.6937	322
Total	1.7235	7.2312	0.8142	27.4308	21.1826	13.9905	23.1223	4.5050	100.0001				
SE	0.0478	0.3396	0.1145	0.5810	0.6465	0.6365	0.5261	0.3276					
PA	96 (2)	76 (3)	80 (10)	82 (1)	85 (2)	74 (3)	90 (1)	82 (4)					
PAa	98 (1)	84 (3)	83 (9)	88 (1)	95 (1)	88 (2)	93 (1)	90 (4)					
n	120	556	95	895	476	369	483	251					3245

**Table 8.** Comparison of UA and PA for the single-date NLCD2019 2019 with (SE) for sample pixels with ca. 2019 Google Earth^™^ reference imagery and without ca. 2019 Google Earth^™^ reference imagery. UA and PA are based on match between the map label and either the primary or alternate reference label.

	UA		PA	

Class	With	Without	With	Without
11	100 (0)	99 (0)	97 (2)	100 (0)
21	87 (4)	93 (5)	53 (5)	77 (8)
22	68 (5)	72 (11)	88 (3)	89 (6)
23	68 (5)	38 (13)	80 (6)	98 (2)
24	74 (4)	45 (16)	81 (7)	100 (0)
31	77 (7)	74 (6)	82 (14)	83 (13)
41	83 (4)	91 (4)	81 (3)	87 (4)
42	95 (2)	92 (3)	85 (3)	90 (3)
43	74 (5)	74 (7)	68 (6)	67 (9)
52	89 (3)	93 (2)	92 (2)	96 (1)
71	83 (4)	92 (2)	87 (4)	86 (3)
81	75 (5)	86 (6)	76 (5)	82 (5)
82	95 (2)	94 (2)	94 (2)	95 (2)
90	57 (7)	63 (7)	92 (3)	88 (6)
95	61 (6)	65 (7)	82 (10)	55 (11)
OA	74.4 (1.4)	80.6 (1.4)		
OAa	84.3 (1.1)	89.8 (1.0)		

**Table 9.** Binary (no change/change) agreement between map and reference labels. See Table 4 for an explanation of contents. OA = 83.1% ± 0.9%; OAa = 90.5% ± 0.7%.

A	Reference						
Map	No Change	Change	Total	UA (SE)	UAa (SE)	MB	n
No Change	95.8437	1.4924	97.3361	98.5 (0.3)	98.5 (0.3)	−0.0802	2127
Change	1.5726	1.0913	2.6639	41.0 (2.2)	48.0 (2.2)	0.0802	1118
Total	97.4163	2.5837	100.0000				
TSE	0.3028	0.3028					
PA (SE)	98.4 (0.1)	42.2 (5.0)					
PAa (SE)	98.6 (0.1)	46.1 (5.1)					
n	2754	491					3245

**Table 10.** Comparison of UA and PA for loss, gain, and no change themes for NLCD2019 versus NLCD2016. Non-overlapping standard errors (SE) were attributed as substantial increase (↑) or decrease (↓). Agreement is based on a match between the map label and either the primary or alternate reference label.

	NLCD2016		NLCD2019			
	2011 – 2016 change		2016 – 2019 change		Substantial	
Themes	UA (SE)	PA (SE)	UA (SE)	PA (SE)	UA	PA
Water loss	46 (9)	77 (11)	28 (6)	49 (21)	↓	↓
Water gain	60 (14)	60 (16)	64 (6)	95 (3)	-	↑
Urban gain	64 (3)	51 (20)	47 (4)	13 (7)	↓	↓
Forest loss	74 (3)	75 (8)	71 (3)	54 (11)	-	-
Forest gain	35 (3)	52 (9)	30 (3)	36 (9)	-	-
Shrubland loss	27 (3)	48 (9)	38 (4)	46 (11)	↑	-
Shrubland gain	29 (3)	59 (9)	36 (4)	58 (11)	↑	-
Grassland loss	34 (3)	80 (11)	37 (4)	71 (11)	-	-
Grassland gain	52 (3)	73 (10)	71 (4)	72 (11)	↑	-
Agriculture loss	27 (3)	20 (12)	23 (4)	10 (9)	-	-
Agriculture gain	33 (3)	23 (8)	14 (3)	13 (12)	↓	↓
Water no Δ	98 (1)	96 (2)	100 (0)	100 (0)	↑	↑
Urban no Δ	94 (1)	83 (3)	94 (1)	87 (3)	-	-
Forest no Δ	96 (1)	90 (1)	94 (1)	91 (1)	-	-
Shrub no Δ	86 (1)	96 (1)	92 (2)	96 (1)	↑	-
Grass no Δ	90 (1)	94 (1)	88 (2)	92 (2)	-	-
Agriculture no Δ	94 (1)	96 (1)	92 (1)	95 (1)	↓	-

**Table 11.** Urban (U) change accuracy. Values of (0) for Standard Error (SE) are less than 0.5%.

	Reference								
Map	U loss	U gain	U no Δ	Never U	Total	UA (SE)	UAa (SE)	MB	n
U loss									
U gain		0.0183	0.0199	0.0123	0.0506	36 (4)	36 (4)	−0.0535	127
U no Δ		0.0137	5.4649	0.4794	5.9580	92 (2)	94 (1)	−1.2270	482
Never U		0.0720	1.7002	92.2192	93.9914	98 (0)	99 (0)	1.2804	2636
Total		0.1041	7.1850	92.7110	100.0000				
TSE		0.0655	0.3365	0.3416					
PA (SE)		18 (11)	76 (3)	99 (0)					
PAa (SE)		57 (18)	86 (3)	100 (0)					
n		61	555	2629					3245

**Table 12.** Forest (F) change accuracy. Values of (0) for Standard Error (SE) are less than 0.5%.

	Reference								
Map	F loss	F gain	F no Δ	Never F	Total	UA (SE)	UAa (SE)	MB	n
F loss	0.3360	0.0063	0.0081	0.0676	0.4909	68 (3)	71 (3)	−0.1860	262
F gain	0.0029	0.1421	0.4293	0.0783	0.6526	22 (3)	22 (3)	0.0219	225
F no Δ	0.2005	0.1660	21.9237	2.1516	24.4418	90 (1)	94 (1)	−2.3556	425
Never F	0.1402	0.3163	4.3634	69.5948	74.4147	94 (1)	97 (0)	2.5224	2333
Total	0.6769	0.6307	26.7974	71.8923	100.0000				
TSE	0.1326	0.1770	0.5855	0.5908					
PA (SE)	49 (10)	23 (7)	82 (1)	97 (0)					
PAa (SE)	53 (10)	57 (17)	91 (1)	97 (0)					
n	204	68	692	2281					3245

## Data Availability

The data are available on the U.S. EPA environmental dataset gateway at https://edg.epa.gov/EPADataCommons/Public/ORD/EnviroAtlas/NLCD2019_AA_RefData.zip. The zipped file includes the requisite ESRI Arc shapefiles and three kmz files that can be used for display in Google Earth^™^. The three kmz files are the reference point (pixel center) locations, reference pixel boundary, and the boundaries of the 3 × 3-pixel neighborhood surrounding the reference pixel. The kmz point file includes reference and map labels and associated attributes. The shapefiles are in an Albers conic equal area projection. Projection parameters and attribute descriptions are in the associated xml files.

## References

[R1] Bianchetti RA, and MacEachren AM. 2015. “Cognitive Themes Emerging from Air Photo Interpretation Texts Published to 1960.” ISPRS International Journal of GeoInformation 4 (2): 551–571. doi:10.3390/ijgi4020551.

[R2] Bianchetti RA, and MacEachren AM. 2015. “Cognitivie Themes Emergin from Air Photo Interpretation Texts Published to 1960.” ISPRS International Journal of GeoInformation 4: 551–571. doi:10.3390/ijgi.4020551.

[R3] Boryan C, Yang Z, Mueller R, and Craig M. 2011. “Monitoring US Agriculture: The US Department of Agriculture, National Agricultural Statistics Service, Cropland Data Layer Program.” Geocarto International 26 (5): 341–358. doi:10.1080/10106049.2011.562309.

[R4] Brown JF, Tollerud HJ, Barber CP, Zhou Q, Dwyer JL, Vogelmann JE, Loveland TR, 2020. “Lessons Learned Implementing an Operational Continuous United States National Land Change Monitoring Capability: The Land Change Monitoring, Assessment, and Projection (LCMAP) Approach.” Remote Sensing of Environment 238: 111356. doi:10.1016/j.rse.2019.111356.

[R5] Cochran WG 1977. Sampling Techniques. 3rd ed. New York: John Wiley & Sons.

[R6] EPA. 2022. “Inventory of U.S. Greenhouse Gas Emissions and Sinks: 1990–2020.” U.S. Environmental Protection Agency, EPA 430-R-22–003. https://www.epa.gov/system/files/documents/2022-04/us-ghg-inventory-2022-main-text.pdf

[R7] FGDC (Federal Geographic Data Committee). 2021. “2020 Lead Cover Agency, NGDA Theme Annual Performance Report for Land Use – Land Cover Theme Geospatial Data Act of 2018, Section 756 Requirements.” https://www.fgdc.gov/gda/gda-lca-theme-reports/fy2020-land-use-land-cover-theme-gda-annual-report.pdf

[R8] Flood N (2013). Seasonal Composite Landsat TM/ETM+ Images Using the Medoid (A Multi-Dimensional Median). Remote Sensing, 5(12), 6481–6500. 10.3390/rs5126481

[R9] Earth Google. n.d. “How Images are Collected.” Accessed January 4 2023. https://support.google.com/earth/answer/6327779?hl=en

[R10] Gopal S, and Woodcock C. 1994. “Theory and Methods for Accuracy Assessment of Thematic Maps Using Fuzzy Sets.” Photogrammetric Engineering & Remote Sensing 60: 181–188.

[R11] Hansen MC, and Loveland TR. 2012. “A Review of Large Area Monitoring of Land Cover Change Using Landsat Data.” Remote Sensing of Environment 122: 66–74. doi:10.1016/j.rse.2011.08.024.

[R12] Holben BN 1986. “Characteristics of Maximum-Value Composite Images from Temporal AVHRR Data.” International Journal of Remote Sensing 7 (1): 417–1434. doi:10.1080/01431168608948945.

[R13] Homer C, Dewitz J, Jin S, Xian G, Costello C, Danielson P, Gass L, 2020. “Conterminous United States Land Cover Change Patterns 2001 – 2016 from the 2016 National Land Cover Database.” ISPRS Journal of Photogrammetry and Remote Sensing 162: 184–199. doi:10.1016/j.isprsjprs.2020.02.019. 35746921 PMC9214659

[R14] Jin S, Dewitz J, Danielson P, Granneman B, Costello C, Smith K, and Zhu Z. in press. “National Land Cover Database 2019: A New Strategy for Creating Clean Leaf-On and Leaf-Off Image Composites.” Journal of Remote Sensing. doi:10.34133/remotesensing.0022.

[R15] Jin S, Homer C, Yang L, Danielson P, Dewitz J, Li C, Zhu Z, Xian G, and Howard D. 2019. “Overall Methodology Design for the United States National Land Cover Database 2016 Products.” Remote Sensing 11 (24): 297. doi:10.3390/rs11242971.

[R16] Lark TJ, Schelly IH, and Gibbs HK. 2021. “Accuracy, Bias, and Improvements in Mapping Crop and Cropland Across the United States Using the USDA Cropland Data Layer.” Remote Sensing 13 (5): 968. doi:10.3390/rs13050968.

[R17] Loveland TR, and Shaw DM (1996) Multiresolution Land Characterization: Building Collaborative Partnerships. In. GAP Analysis: A Landscape Approach to Biodiversity Planning (edited by Scott JM, Tear T, and Davis F), Proceedings of the ASPRS/GAP Symposium, Charlotte, NC (National Biological Service, Charlotte, pp. 83–89.

[R18] Olofsson P, Arévalo P, Espejo AB, Green C, Lindquist E, McRoberts RE, and Sanz MJ. 2020. “Mitigating the Effects of Omission Errors on Area and Area Change Estimates.” Remote Sensing of Environment 236: 111492. doi:10.1016/j.rse.2019.111492.

[R19] Olofsson P, Foody GM, Herold M, Stehman SV, Woodcock CE, and Wulder MA. 2014. “Good Practices for Estimating Area and Assessing Accuracy of Land Change.” Remote Sensing of Environment 148: 42–57. doi:10.1016/j.rse.2014.02.015.

[R20] Pengra BW, Stehman SV, Horton JA, Dockter DJ, Schroeder TA, Yang Z, Cohen WB, Healy SP, and Loveland TR. 2020. “Quality Control and Interpreter Consistency of Annual Land Cover Reference Data in an Operation National Monitoring Program.” Remote Sensing of Environment 238: 111261. doi:10.1016/j.rse.2019.111261.

[R21] Qui S, Zhu S, Olofsson P, Woodcock CE, and Jin S. 2023. “Evaluation of Landsat Image Compositing Algorithms.” Remote Sensing of Environment. doi:10.1016/j.rse.2022.113375.

[R22] Roy DP, Ju J, Kline K, Scaramuzza PL, Kovalskyy V, Hansen M, Loveland TR, Vermote E, and Zhang C. 2010. “Web-Enabled Landsat Data (WELD): Landsat ETM+ Composited Mosaics of the Conterminous United States.” Remote Sensing of Environment 114 (1): 35–49. doi:10.1016/j.rse.2009.08.011.

[R23] Särndal CE, Swensson B, and Wretman J. 1992. Model-Assisted Survey Sampling. New York: Springer-Verlag.

[R24] SAS Institute Inc. 2017. SAS/STAT^®^ 14.3 User’s Guide. Cary, NC.

[R25] Stehman SV 2001. “Statistical Rigor and Practical Utility in Thematic Map Accuracy Assessment.” Photogrammetric Engineering & Remote Sensing 67: 727–734.

[R26] Stehman SV (2014). Estimating Area and Map Accuracy for Stratified Random Sampling When the Strata are Different from the Map Classes. International Journal of Remote Sensing, 35(13), 4923–4939. 10.1080/01431161.2014.930207

[R27] Stehman SV, and Czaplewski RL. 1998. “Design and Analysis for Thematic Map Accuracy Assessment: Fundamental Principles.” Remote Sensing of Environment 64 (3): 331–344. doi:10.1016/S0034-4257(98)00010-8.

[R28] Stehman SV, and Foody GM. 2019. “Key Issues in Rigorous Accuracy Assessment of Land Cover Products.” Remote Sensing of Environment 231: 111199. doi:10.1016/j.rse.2019.05.018.

[R29] Stehman SV, and Wickham JD. 2011. “Pixels, Blocks of Pixels, and Polygons: Choosing a Spatial Unit for Thematic Accuracy Assessment.” Remote Sensing of Environment 115 (12): 3044–3055. doi:10.1016/j.rse.2011.06.007.

[R30] Stehman SV, Wickham JD, Smith JH, and Yang L. 2003. “Thematic Accuracy of the 1992 National Land-Cover Data (NLCD) for the Eastern United States: Statistical Methodology and Regional Results.” Remote Sensing of Environment 86 (4): 500–516. doi:10.1016/S0034-4257(03)00128-7.

[R31] USGS. 2021. “Landsat 4–7 Collection 2 (C2) Level 2 Science Product (L2SP) Guide. Version 4.” U.S. Geological Survey, LSDS-1618, September. https://d9-wret.s3.us-west-2.amazonaws.com/assets/palladium/production/s3fs-public/media/files/LSDS-1618_Landsat-4-7_C2-L2-ScienceProductGuide-v4.pdf

[R32] USGS. 2022. “Landsat 8–9 Collection 2 (C2) Level 2 Science Product (L2SP) Guide. Version 4.” U.S. Geological Survey, LSDS-1619, March. https://d9-wret.s3.us-west-2.amazonaws.com/assets/palladium/production/s3fs-public/media/files/LSDS-1619_Landsat-8-9-C2-L2-ScienceProductGuide-v4.pdf

[R33] USGS. n.d. “National Land Cover Database Class Legend and Description.” U.S. Geological Survey. Accessed January 4 2023. https://www.mrlc.gov/data/legends/national-land-cover-database-class-legend-and-description

[R34] Van Coillie FMB, Gardin S, Anseel F, Duyck W, Verbeke LPC, and De Wulf RR. 2014. “Variability of Operator Performance in Remote Sensing Image Interpretation: The Importance of Human and External Factors.” International Journal of Remote Sensing 35 (2): 754–778. doi:10.1080/01431161.2013.873152.

[R35] Wickham J, Homer C, Vogelmann J, McKerrow A, Mueller R, Herold N, and Coulston J. 2014. “The Multi-Resolution Land Characteristics (MRLC) Consortium — 20 Years of Development and Integration of USA National Land Cover Data.” Remote Sensing 6: 7424–7441. doi:10.3390/rs6087424.

[R36] Wickham JD, Stehman SV, Fry JA, Smith JH, and Homer CG. 2010. “Thematic Accuracy of the NLCD 2001 Land Cover for the Conterminous United States.” Remote Sensing of Environment 114 (6): 1286–1296. doi:10.1016/j.rse.2010.01.018.

[R37] Wickham J, Stehman SV, Gass L, Dewitz J, Fry JA, and Wade TG. 2013. “Accuracy Assessment of NLCD 2006 Land Cover and Impervious Surface.” Remote Sensing of Environment 130: 294–304. doi:10.1016/j.rse.2012.12.001.

[R38] Wickham J, Stehman SV, Gass L, Dewitz JA, Sorenson DG, Granneman BJ, Poss RV, and Baer LA. 2017. “The Accuracy Assessment of the 2011 National Land Cover Database (NLCD).” Remote Sensing of Environment 191: 328–341. doi:10.1016/j.rse.2016.12.026. 31346298 PMC6657805

[R39] Wickham J, Stehman SV, Sorenson DG, Gass L, and Dewitz JA. 2021. “Thematic Accuracy of the NLCD 2016 Land Cover for the Conterminous United States.” Remote Sensing of Environment 257: 112357. doi:10.1016/j.rse.2021.112357. 39749320 PMC11694894

[R40] Wulder MA, Loveland TR, Roy DP, Crawford CJ, Masek JG, Woodcock CE, Allen RG, 2019. “Current Status of Landsat Program, Science, and Applications.” Remote Sensing of Environment 225: 127–147. doi:10.1016/j.rse.2019.02.015.

[R41] Yang L, Jin S, Danielson P, Homer C, Gass L, Bender SM, Case A, 2018. “A New Generation of the United States National Land Cover Database: Requirements, Research Priorities, Design, and Implementation Strategies.” ISPRS Journal of Photogrammetry and Remote Sensing 146: 108–123. doi:10.1016/j.isprsjprs.2018.09.006.

